# Coupled cell networks are target cells of inflammation, which can spread between different body organs and develop into systemic chronic inflammation

**DOI:** 10.1186/s12950-015-0091-2

**Published:** 2015-07-25

**Authors:** Elisabeth Hansson, Eva Skiöldebrand

**Affiliations:** Department of Clinical Neuroscience and Rehabilitation, Institute of Neuroscience and Physiology, The Sahlgrenska Academy, University of Gothenburg, Per Dubbsgatan 14, 1tr, , SE 413 45 Gothenburg, Sweden; Section of Pathology, Department of Biomedical Sciences and Veterinary Public Health, Swedish University of Agricultural Sciences, Uppsala, Sweden; Department of Clinical Chemistry and Transfusion Medicine, Institute of Biomedicine, Sahlgrenska University Hospital, Gothenburg University, Gothenburg, Sweden

**Keywords:** Coupled cell networks, Inflammation, Pain spreading, Ca^2+^ signaling, Connexin 43, Gap junctions

## Abstract

Several organs in the body comprise cells coupled into networks. These cells have in common that they are excitable but do not express action potentials. Furthermore, they are equipped with Ca^2+^ signaling systems, which can be intercellular and/or extracellular. The transport of small molecules between the cells occurs through gap junctions comprising connexin 43. Examples of cells coupled into networks include astrocytes, keratinocytes, chondrocytes, synovial fibroblasts, osteoblasts, connective tissue cells, cardiac and corneal fibroblasts, myofibroblasts, hepatocytes, and different types of glandular cells. These cells are targets for inflammation, which can be initiated after injury or in disease. If the inflammation reaches the CNS, it develops into neuroinflammation and can be of importance in the development of systemic chronic inflammation, which can manifest as pain and result in changes in the expression and structure of cellular components. Biochemical parameters of importance for cellular functions are described in this review.

## Introduction

### Inflammation and neuroinflammation

In conditions that lead to inflammation, changes in several cellular parameters of coupled cell networks occur throughout many organs in the body. Inflammation is a physiological response to injury that is designed to remove dangerous stimuli, kill bacteria and viruses, remove cell debris, and initiate healing. It can persist or become exaggerated and may cause undesirable negative effects. Inflammation can be induced by several substances produced or released by tissues or by environmental factors, including circulating glucose, gut microflora, interleukins, endotoxins or other toxins, all of which have an impact on immune receptor expression. The underlying molecular processes are just beginning to be elucidated.

When an injury occurs in peripheral tissue, pro-inflammatory mediators are released into the bloodstream, and white blood cells are attracted to the injury site. The endothelium lining the blood vessels becomes permeable, allowing leukocytes to migrate from the blood vessels to the injury site [1, 2]. The pro-inflammatory mediators released can increase the permeability of the blood–brain barrier (BBB), leading to the passage of blood cells into the central nervous system (CNS) [3, 4]. This process is known as neuroinflammation [5]. These blood cells are transformed into reactive microglia, which produce pro-inflammatory cytokines and activate astrocytes. This combined response causes a change in astrocyte network signaling, which is involved in monitoring neuronal signaling as well as rebuilding synapses [6].

Neuroinflammation can also be initiated when a local peripheral injury gives rise to inflammatory activation in the CNS at the site of the damaged or affected nerve(s) [7–9]. The inflammatory cascade is activated, and immunocompetent cells migrate to the site of injury. Such cells can be mast cells, which are capable of migrating across the BBB in situations where the barrier is compromised as a result of CNS pathology [10]. Pericytes in the microvessels respond to immune activation and may play an important role in communicating inflammatory signals [11]. Myofibroblasts, developed from fibroblasts and maybe also from pericytes, are considered to be the dominant collagen-producing cells and are activated when structural and functional defects occur [12]. As a result, the subsequent neuroinflammatory environment causes the activation of glial cells located in the dorsal horn of the spinal cord. Macrophages infiltrate the injured nerve and cause an inflammatory reaction in the neurons [13], which leads to microglial activation in the CNS and pro-inflammatory cytokine release. These cytokines then activate and alter astrocyte function [9, 14]. Once the astrocytes and microglia have been activated, they participate in the development, spread, and potentiation of neuroinflammation [15, 16], resulting in low-grade inflammation [7] along the pain pathways from the periphery to the spinal cord, extending up to the thalamus and farther onto the parietal cortex.

If this dysfunction persists for a long time, it can lead to pathogenic chronic neuroinflammation and can transition into long-term pain [8, 9, 17]. When an inflammatory response is activated throughout the body, the event can affect non-lesioned structures on both the ipsilateral and contralateral sides [18].

### Coupled cell networks

**Fig. 1:**
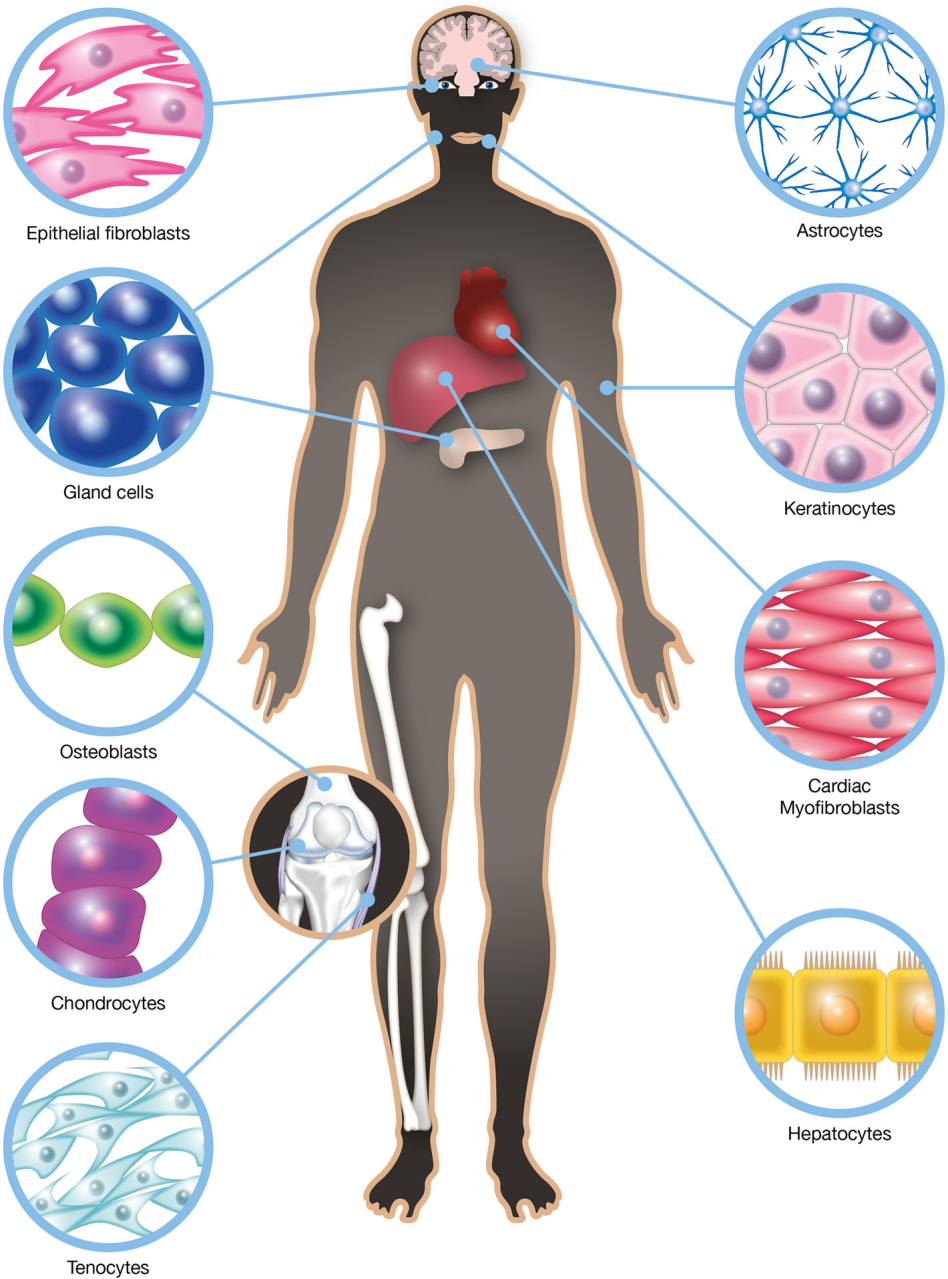
Schematic illustration highlighting the different organs in the body that comprise cells coupled into networks. These cells are excitable but do not express action potentials. They are equipped with Ca^2+^ signaling systems, and the transport of small molecules between the cells occurs through gap junctions. Examples of cells coupled into networks are astrocytes in the brain, keratinocytes in the skin and buccal membranes, chondrocytes in the articular cartilage, osteoblasts in bone, connective tissue cells such as epithelial cells in the cornea and tenocytes in the ligaments, cardiac fibroblasts in the heart, hepatocytes in the liver, and different types of glandular cells throughout the body. The illustration was created by Pontus Andersson, ArtProduction, Gothenburg, Sweden

**Fig. 2:**
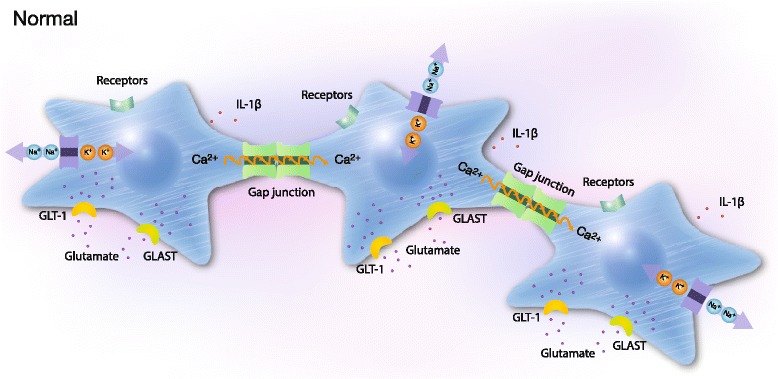
Schematic illustration of three cells coupled in networks under physiological conditions. The cells are coupled by gap junctions to form extensive cellular networks. Receptors, ion pumps, glutamate transporters, actin filaments (white bands), cytokine release and Ca^2+^ signalingare shown. Cytosolic Ca^2+^ plays key roles as a second messenger and a sensor of Ca^2+^-dynamics in cell networks and detects changes in the microenvironment. Two communication pathways exist: the major intercellular via gap junctions comprising Cx43. Receptors on the surface of cells are coupled to G proteins and release Ca^2+^ from the endoplasmic reticulum via PLC and IP_3_. Ca^2+^ elevations are followed by the propagation of intercellular Ca^2+^ waves through the gap junctions, which are also permeable to molecules less than 1.5 kDa such as Ca^2+^, IP_3_, and cAMP. The minor extracellular pathway involves ATP release through hemichannels. The illustration was created by Pontus Andersson, ArtProduction, Gothenburg, Sweden

Similarities exist between different types of coupled cell networks in different body organs with respect to several cellular parameters. Examples of cells coupled into networks include astrocytes, keratinocytes, chondrocytes, synovial fibroblasts, osteoblasts, connective tissue cells, cardiac and corneal fibroblasts, myofibroblasts, hepatocytes, and different types of glandular cells (Fig. 1). Intercellular communication gives tissues the ability to coordinate many cellular functions such as the regulation of cell volume, intracellular ionic composition, and cell metabolism. Characteristics such as their passive electrical properties not only provide the framework and metabolic support for different organs but also contribute to their computational power and behavioral output. These properties enable more active functions and are endowed through Ca^2+^-based excitability [19]. Intracellular Ca^2+^ changes are important due to their influence on many cell functions, including matrix synthesis and degradation [20]. An increase in cytosolic Ca^2+^ levels can lead to the release of signaling molecules such as transmitters, cytokines, prostaglandins, proteins, and peptides via regulated exocytosis [21]. The dynamic components of exocytosis include the vesicular-plasma membrane secretory machinery and vesicular traffic, which is governed by general cytoskeletal elements [22]. For this machinery to work, intercellular structures called gap junctions, which directly connect the interior of adjacent cells through a pathway not open to the extracellular space, appear necessary [23]. Gap junction channels comprise two hemichannels, called connexons, one of which is provided by each of the joined cells. These channels select for the direct exchange of ions, metabolites, and small molecules such as Ca^2+^, adenosine triphosphate (ATP), nicotinamide adenine dinucleotide (NAD^+^), glutamate, prostaglandins, and glutathione, which are less than 1.5 kDa in size, between contiguous cells [24]. Connexin 43 (Cx43) is the primary gap junction protein [25]. Cytoskeletal reorganization is pivotal event in all of these processes; dynamic remodeling of the actin cytoskeleton plays an essential role in cell migration and proliferation. Actin appears in two forms, globular actin (G-actin) and filamentous actin (F-actin), and the transition between these two forms is a dynamic process driven by polymerization and depolymerization [26] (Fig. 2).

## Coupled cell networks in different body organs

### Astrocytes

The well-studied cells coupled into networks are astrocytes in the CNS [6, 21]. Astrocytes in networks positioned between the vasculature and synapses monitor neuronal signaling and synapse rebuilding [6]. Astrocytes express nearly the same repertoire of receptors and ion channels as neurons, regulate synaptic transmission via bidirectional communication with neurons, and release gliotransmitters and other factors such as cytokines, fatty acid metabolites, and free radicals [27, 28].

Because they do not communicate via action potentials, astrocytes are not electrically active; however, they display a form of excitability that manifests as an increased intracellular Ca^2+^ concentration. Stimuli such as transmitters released from neurons and glial cells can evoke Ca^2+^ elevation in single astrocytes, which passes to adjacent astrocytes and leads to a Ca^2+^ wave that can propagate over long distances, albeit much more slowly than the propagation of action potentials in neurons [19, 29, 30].

Incoming stimuli activate G protein-coupled receptors that hydrolyze phosphatidylinositol 4,5-bisphosphate (PIP_2_) and cause the release of inositol 1,4,5-trisphosphate (IP_3_) into the cytosol. IP_3_ receptors located on the endoplasmic reticulum respond to this elevation of IP_3_ by releasing Ca^2+^. Cytosolic Ca^2+^ plays a key role as a second messenger; thus, the control of Ca^2+^ signals is critical. This control involves coordinating Ca^2+^ entry across the plasma membrane, Ca^2+^ release from the endoplasmic reticulum, endoplasmic reticulum store refilling, and Ca^2+^ extrusion across the plasma membrane [31]. The Na^+^-Ca^2+^ exchanger, a Ca^2+^ transporter that controls the intracellular Ca^2+^ concentration, is driven by the Na^+^ electrochemical gradient across the plasma membrane.This Na^+^ pump, the Na^+^/K^+^-ATPase, indirectly modulates Ca^2+^ signaling [32], and inflammatory stimuli influence Ca^2+^ homeostasis in astrocyte networks [33–35].

Astrocytes are directly connected to adjacent cells by gap junctions, and Cx43 is the primary gap junction protein [25]. Astrocytes also express hemichannels that open exteriorly; Cx43 appears to also be the main Cx found in these hemichannels [24]. Astrocytes in most parts of the CNS use two types of Ca^2+^ communication: intercellular communication through gap junctions and extracellular communication through the diffusion of ATP, which then binds to purinoceptors. Both inter- and extracellular Ca^2+^ communication occur in many parts of the cerebrum [19, 36]. In the retina, intercellular communication occurs through astrocytes, but extracellular communication occurs between astrocytes and Müller cells [37].

The cytoskeleton is important for controlling plasma membrane microdomains and the endoplasmic reticulum complex. The adaptor protein ankyrin B is associated with the Na^+^ pump as well as with endoplasmic reticulum proteins such as IP_3_. The primary cytoplasmic matrix proteins spectrin and actin are attached to ankyrin B. An intact cytoskeleton is required for astrocytic Ca^2+^ wave propagation [36], and cytoskeletal disruption abolishes Ca^2+^ oscillations by changing the balance of the Ca^2+^ regulatory processes [38].

Astrocytes contribute to the homeostasis and regulation of extracellular glutamate levels. The glutamate-glutamine cycle is a well-known process through which glutamine is released from astrocytes and taken up by glutamatergic or γ-aminobutyric acid (GABA)ergic neurons. Glutamine is then converted to glutamate in neurons and released into the synaptic space. The majority of the released glutamate is taken up by astrocytes through the glutamate transporters; glutamate/aspartate transporter (GLAST/excitatory amino acid transporter 1, EAAT1), and glial glutamate transporter-1 (GLT-1/EAAT2) and then metabolized by glutamine synthetase to glutamine [39, 40].

### Keratinocytes

The epidermis is a dynamic, stratified structure formed by continually proliferating and differentiating keratinocytes that surround the sensory nerve endings of several C- and Aδ-fiber subtypes. The skin and buccal membrane primarily comprise keratinocytes, the epidermis. The cells are connected by well-developed intercellular junctions such as gap junctions. Within these gap junctions, Cx43 is associated with the regulation of cell proliferation and mediates forms of intercellular communication in which ions and small molecules are allowed to pass from one cell to another. Cx43 is primarily localized to the lower epithelial layer, the stratum basale and stratum spinosum [41]. Cx43 degradation is thought to play a role in the differentiation of the gingival epithelium. Properly regulated gap junctions appear to be essential for efficient wound healing and for protection against skin diseases. Human epidermal keratinocytes use intercellular Ca^2+^ signaling. G protein-coupled receptors, which activate phospholipase C (PLC) and convert PIP_2_ into diacylglycerol and IP_3_, trigger the release of Ca^2+^ from intracellular Ca^2+^ stores [42, 43].

In response to stress, injury or even chronic pain, keratinocytes can release ATP through hemichannels, resulting in Cx43 upregulation. Metabotropic purinergic (P2Y_2_) receptors are then activated, resulting in increased intracellular Ca^2+^ [44]. ATP release is an important signal for epidermal homeostasis and influences keratinocyte proliferation and differentiation [45]. Glutamate-mediated signaling is observed in keratinocytes in the epidermis, and different classes of glutamate receptors, including N-methyl-D-aspartate receptor (NMDA), α-amino-3-hydroxy-5-methyl-4-isoxazolepropionic acid receptor (AMPA) and metabotropic glutamate receptors, as well as transporters such as EAAT1 have been identified in the basal layer. Additionally, GLT-1 has been found in the suprabasal layer [46].

### Chondrocytes

Chondrocytes are connected to each other via cell-to-cell interactions and form functional gap junctions that express Cx43 [47, 48]. They can sustain the propagation of intercellular Ca^2+^ waves in rabbits [47], humans, and equines [49, 50] and can also form hemichannels that exchange signals within the extracellular space [51] (Skiöldebrand et al., unpublished). Articular chondrocytes accumulate intracellular IP_3_ following mechanical stimulation, causing the diffusion of IP_3_ into adjacent cells through gap junctions and amplification of the response. In adult articular cartilage, chondrocytes exist as individual cells embedded in the extracellular matrix, and gap junctions are mainly expressed by the flattened chondrocytes facing the outer cartilage layer where intercellular communication occurs [52]. The role of Cx43 in chondrocytes has not been extensively studied, but Cx43 is required for the differentiation and metabolic homeostasis of the extracellular matrix [48]. Cx43 also functions as a hemichannel to release ATP and NAD^+^. Chondrocytes express purinergic receptors such as P2-purinoceptors that induce intracellular Ca^2+^ responses. These intracellular Ca^2+^ responses are increased following stimulation with IL-1 [53]. Glutamate and substance P have been identified in human articular chondrocytes. Neurokinin 1 (NK1) and glutamate receptors are also expressed, as well as both metabotropic and ionotropic glutamate receptors and the glutamate transporters GLT-1 and GLAST [54].

### Bone cells

Gap junctional communication plays a critical role in the coordination of bone remodeling. The bone-forming cells osteoblasts and osteocytes primarily express Cx43 but also express Cx45 and Cx46, which form functional gap junctions [55]. Cx43 expression increases during differentiation, and inhibition of this communication leads to retardation of the differentiation process, resulting in a reduced ability to form mineralized extracellular matrix. Through mechanical manipulation, the osteoblasts, which are non-excitable, produce synchronized Ca^2+^ waves, which involve the release of IP_3_-sensitive intracellular Ca^2+^ stores. These waves occur either via gap junction-mediated intercellular Ca^2+^ signaling or as a result of the autocrine activity of released ATP, which stimulates P2 purinoceptors. The P2Y class comprises G protein-coupled receptors that activate PLC, resulting in IP_3_ generation and intracellular Ca^2+^ store release in human osteoblasts [56]. Hemichannels have also been reported in osteoblasts [55].

### Connective tissue cells

Gap junctions are found in tendons, ligaments, synovium (within the synovial membrane), and corneal stroma because the cells of these tissues are coupled to form networks. Two adjacent cells join through Cx43, allowing direct cell-to-cell communication via Ca^2+^ signaling. In osteoarthritis, the synovial fibroblasts produce pro-inflammatory cytokines and catabolic proteases, leading to degradation of the extracellular matrix. The role of Cx43 in osteoarthritis involves an increase in its expression in both chondrocytes and synovial cells, which affects catabolic and pro-inflammatory genes [57]. Tenocytes respond to mechanical signals by transforming them into biochemical signals via a second messenger such as Ca^2+^ or IP_3_ [58]. The mechanical load directly regulates gap junction permeability [59]. Some of the Cxs assemble to form hemichannels [60]. Through mechanical stimulation, ATP is released and acts in a paracrine or autocrine manner through the stimulation of P2Y_2_ purinoceptors, resulting in increased intracellular Ca^2+^ [58]. Cx43 associates with actin to stabilize gap junctions at the plasma membrane [61].

### Cardiac fibroblasts

Cardiac fibroblasts are the most abundant cell type in the heart, play a key role in the myocardial maintenance and repair, and can transform into cardiac myofibroblasts, which are present in valve leaflets in the adult heart. These cells express α-smooth muscle actin (α-SMA) and are referred to as α-SMA-containing stress fibers [62]. The cells are joined by gap junctions that express Cx43 [63], enabling Ca^2+^ signaling that causes the release of Ca^2+^ from the endoplasmic reticulum in response to ATP, histamine, 5-hydroxytryptamine (5-HT) [64] (Lundqvist et al., unpublished), or bradykinin [65]. These cells produce extracellular matrix, exhibit high Na^+^/K^+^-ATPase activity levels in the extracellular matrix [66], and also produce and release a substantial number of cytokines and growth factors into their environment, thereby regulating cell function in an autocrine and paracrine manner [62].

### Hepatocytes

Agonist-evoked Ca^2+^ signals are found in the liver and are manifested as the propagation of intercellular Ca^2+^ waves through liver cells called hepatocytes. Agonist binding to plasma membrane receptors stimulates G_q_ proteins, which activate PLC and cause Ca^2+^ mobilization from internal stores [67]. The intercellular propagation normally takes place through Cx43-containing gap junctions [68]. ATP release into the extracellular space stimulates purinoceptors, a paracrine signaling pathway [69].

### Glandular cells

Intercellular signaling in salivary glands has been observed when 5-HT triggers intercellular Ca^2+^ waves through gap junctions and induces Ca^2+^ release via the IP_3_ receptor [70]. Pancreatic acinar cells in the exocrine part of the gland also conduct intercellular Ca^2+^ signaling between cells [71].

## Inflammation at the cellular level

During inflammation, the expression and affinities of several receptors are changed. In astrocytes, Toll-like receptor 4 (TLR4) expression increases [72, 73], and opioid receptors alter their responses to agonists and antagonists [74, 75].

**Fig. 3:**
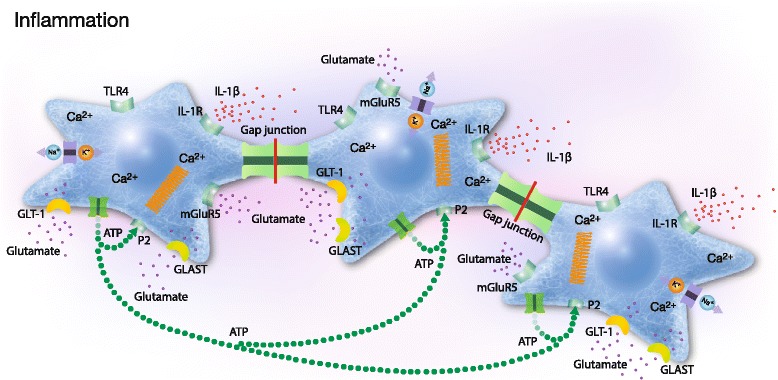
In cells experiencing inflammation, glutamate release into the extracellular space is increased, and Ca^2+^ signaling in cellular networks is over-activated. Several receptors are influenced by the increased expression of TLR4, and the responses of other receptors such as P2 and mGluR5 are also changed. The Na^+^/K^+^-ATPase is downregulated, the glutamate transporters GLT-1 and GLAST are changed, and actin filaments (white bands disorganized) in the cytoskeleton are reorganized, thereby abolishing the balance between the Ca^2+^ regulatory processes. This process leads to the down-regulation of intercellular Ca^2+^ signaling and thereby of Cx43 in gap junctions. ATP is released from the cells and binds to P2 purinoceptors, which generates extracellular Ca^2+^ oscillations/waves and increased release from internal stores. The increased release of pro-inflammatory cytokines such as IL-1β occurs extracellularly. The clearing of increased glutamate concentrations from the extracellular space, which plays a critical role in preventing glutamate excitotoxicity, is attenuated. The illustration was created by Pontus Andersson, ArtProduction, Gothenburg, Sweden

Furthermore, the cytoskeleton is disrupted into more diffuse and ring-structured actin filaments. Lipopolysaccharide (LPS) exposure alters the actin cytoskeleton in astrocytes [73], macrophages [76], neutrophils [77], and pulmonary monocytes [78]. Ca^2+^ signaling in the astrocyte network is elevated, resulting in increased ATP production and release through the opening of hemichannels. ATP stimulates purinoceptors through autocrine or paracrine mechanisms and results in increased Ca^2+^ release from internal stores; this release occurs in the form of Ca^2+^ oscillations, which may change the balance of Ca^2+^-regulating processes [21]. This extracellular Ca^2+^ signaling attenuates intercellular Ca^2+^ signaling, causing reduced communication via gap junctions [79]. Sodium transporters such as the Na^+^/K^+^-ATPase are downregulated [73]. Neuronal excitability is increased due to the inflammation, leading to increased glutamate release at neural synapses. Astrocytes are the predominant players in clearing glutamate from the extracellular space. The uptake of excessive extracellular glutamate by astrocytes via the membrane-bound glutamate transporters GLAST and GLT-1 plays a critical role in preventing glutamate excitotoxicity [80]. Glutamate uptake transporters are downregulated in the presence of excess glutamate, which leads to the inhibition of glutamate uptake [81]. The increased release of pro-inflammatory cytokines such as tumor necrosis factor-α (TNF-α) and IL-1β also occurs [72, 73, 82, 83] (Fig. 3).

Inflammation plays a part in most, if not all, CNS insults. Examples of inflammatory diseases where some of these cellular parameters have been shown to be changed are in the Alzheimer’s disease [84], Parkinson’s disease [85], multiple sclerosis [86], traumatic injury and ischemia, autoimmune inflammation, and damage and diseases where immune and inflammatory cells have crucial roles in these responses [87].

Changes in cellular parameters due to inflammation are also observed in other cellular networks.

The cellular location of TLR4 in keratinocytes has a key role in the initiation of innate immunity and in the regulation of adaptive immune responses. TLR4 may also be an important regulator of wound inflammation [88] and dermal wound healing [89]. Keratinocytes play a role in the pathogenesis of cutaneous inflammatory disease by producing pro-inflammatory cytokines such as IL-1α, IL-1β, TNF-α, IL-6, IL-8 and granulocyte-macrophage colony-stimulating factor, adhesion factors, and co-stimulatory molecules [90].

In rheumatoid arthritis, the synovial lining cells exhibit an infiltration of macrophages, increased levels of pro-inflammatory cytokines, and upregulation of catabolic matrix-degrading enzymes such as matrix metalloproteinases, which leads to the activation and destruction of cartilage and bone cells. An interaction between LPS, TLR4, and collagen type II in chondrocytes has a role in initiating this pro-inflammatory activity and can lead to the inhibition of cartilage extracellular matrix production and chondrocyte inflammation and apoptosis [91]. The stimulation of chondrocytes with IL-1β causes the significant up-regulation of TLR4 [92], increased production of metalloproteinases, suppression of type II collagen and proteoglycan production and induction of pro-inflammatory mediators such as prostaglandins and nitric oxide [49]. The degradation of articular cartilage is a characteristic feature of arthritic diseases, and IL-1 is one of the more potent cytokines that promotes cartilage catabolism, enhances Cx43 expression and increases Ca^2+^ signaling [47]. Altered Cx43 expression may be an early phenotypic event in osteoarthritis [48]. The concentration of glutamate in synovial fluid is notably increased in both osteoarthritis (a low-grade inflammatoryjoint disease) and rheumatoid arthritis (a chronic autoimmune disease) and is correlated with increased inflammatory mediators such as TNF-α and chemokines [93].

The increased extracellular glutamate concentration is regulated by glutamate transporters in rat chondrocytes [94] as well as in human cartilage [54]. Glutamate may function as an autocrine factor. Ionotropic glutamate receptors contribute to pain and the inflammatory stages of osteoarthritis, and receptor antagonists have been proposed as a potential treatment [95]. Adenosine receptors have important roles in the regulation of inflammation and may be involved in the inhibition of pro-inflammatory cytokine release, especially in chondrocytes and osteoblasts [96].

Cardiac fibroblasts likely have important roles in the inflammatory process in the heart. These cells transform into myofibroblasts in conditions of reduced ventricular function; extracellular matrix production is increased, chemokine production is upregulated, and inflammatory pathways are changed. Furthermore, these fibroblasts recruit monocytes into cardiac tissue [97]. Mechanical stress, which also induces increased chemokine production, appears to be important in the inflammatory process.

During pathophysiological inflammation in salivary gland cells, LPS induces the secretion of IL-β into the saliva. TLR4 is upregulated via the LPS signaling pathway [98]. Inflammation in the mammary gland during lactation leads to TLR4 activation and increased IL-8 secretion [99]. Inflammation in the lacrimal gland results in up-regulation of TLR4 in the corneal epithelium of the eye [100].

TLR4 may be involved in the immune response in liver disease caused by hepatitis B, which can lead to inflammation, cirrhosis, and hepatocellular carcinoma [101].

Airway inflammation is often caused by Gram-negative bacteria or by the presence of endotoxins, which can lead to the development of asthma. TLR4 also seems to play an important role in this process [102].

## Systemic inflammation and the spread of inflammation

Do coupled cell networks in different organs possess a signaling system that can spread or propagate from the coupled cell networks of one organ to those in other organs on either the contralateral or ipsilateral side? Can chronic, low-grade, systemic inflammation influence coupled cell networks in one or several organs, leading to chronic tissue degradation?

Obesity promotes chronic, low-grade, systemic inflammation that contributes to the development of type 2 diabetes, cardiovascular disease and metabolic disorders such as liver steatosis [103, 104]. Both the intestinal microbiota and a high-fat diet have been shown to induce changes in gut homeostasis and consequent mucosal inflammation. Intestinal inflammation may be an early event that leads to the systemic inflammation of several organs [105]. Furthermore, there appear to be strong links between type 2 diabetes, dementia and neurodegenerative diseases such as Alzheimer’s disease. Underlying mechanisms involved seem to be defaults in cellular insulin resistance, inflammatory and oxidative stress pathways [106]. Additional autoimmune rheumatic diseases are thought to result in both joint and systemic inflammation, with TNF-α acting as the prominent cytokine. This process may be an initiator of neuroinflammation [5] and might increase the risk of cardiovascular tissue destruction [107]. Transient receptor potential (TRP) channels, expressed in non-excitable coupled cell networks [108, 109] play important roles in physiological as well as in pathological processes; inflammation and pain. They activate signal pathways via Ca^2+^ entry and membrane depolarization. They contribute to cell volume regulation and are involved in diseases such as osteoarthritis, cardiovascular disorders, type 2 diabetes mellitus etc. [108, 110, 111]. Stimulation of TRP channels can lead to astrocytic reactions followed by activation of nociceptive input [109]. Osteoarthritis is a condition characterized by mainly pain, reduced joint movement and signs of inflammation, such as swelling. TRP channels antagonists have been investigated as a novel therapy by alleviating pain and some inflammatory aspects [112].

Innate immune dysregulation can be the driver for autoinflammatory diseases. It is efforts to define overlapping, maybe genetically determined inflammatory responses in autoimmunity and infection diseases [113]. These questions are important to reflect on, and more studies are required to obtain more conclusive answers.

## Pain processing and mirror pain due to neuroinflammation

CNS glia, especially microglia and astrocytes coupled into networks, appear to be capable of action at a distance. The nervous system can also initiate signals that alter the function of glial cells. Activated glia release immunomodulatory products, which can be key mediators of the modulation of neuronal activity. It may lead to low-grade inflammation at the site of the damaged or affected peripheral nerve(s). Inflammatory receptors are affected, and interactions between these receptors may induce immune signaling changes, which may be of importance in neuroinflammation. Such immune-mediated inflammation can be underlying mechanisms of persistent or long-term pain. Astrocytes and microglia become activated after pain-activating substances are released from neurons located in the spinal cord and brain in response to peripheral and/or CNS trauma, which can lead to over-activation [7] that results in morphological and functional changes. Additionally, sodium transporters down-regulation and cytoskeletal disruption occur, thereby abolishing Ca^2+^ signaling by altering the balance between Ca^2+^-regulating processes [114].

Changes in the release of pro- and anti-inflammatory cytokines are observed in many clinical studies [83, 115]. These mediators can underlie the spread of the neuroinflammation and pain to the uninjured side [8]. Effects on contralateral non-lesioned structures have been well-documented, and these effects also occur on the ipsilateral side. Unidentified signaling mechanisms linking either the same side of the body or the two sides of the body likely exist [18]. Glial cells and potentially astrocytes can participate in the activation and initiation of signals that regulate neuronal function but may also initiate signals in other parts of the body.

## Conclusion

Coupled cell networks throughout the body are coupled by gap junctions expressing Cx43 and Ca^2+^ signaling systems. These cells are targets in conditions that can lead to immune-mediated inflammation. Several basal cellular parameters are then changed. One hypothesis is that coupled cell networks use a signaling mechanism between the cells within their own networks. The signaling can be transferred to other coupled cell networks in other organs. This form of communication may give rise to inflammatory systemic diseases. Such signaling may facilitate the spreading of pain to sites both contralateral and ipsilateral to the original injury site; this spreading is sometimes observed with long-term pain.

## References

[1] Machelska H (2011). Dual peripheral actions of immune cells in neuropathic pain. Arch Immunol Ther Exp (Warsz).

[2] Huang E, Wells CA (2014). The ground state of innate immune responsiveness is determined at the interface of genetic, epigenetic, and environmental influences. J Immunol.

[3] Huber JD, Witt KA, Hom S, Egleton RD, Mark KS, Davis TP (2001). Inflammatory pain alters blood–brain barrier permeability and tight junctional protein expression. Am J Physiol Heart Circ Physiol.

[4] Sharma HS, Johanson CE (2007). Blood-cerebrospinal fluid barrier in hyperthermia. Prog Brain Res.

[5] Fuggle NR, Howe FA, Allen RL, Sofat N (2014). New insights into the impact of neuro-inflammation in rheumatoid arthritis. Front Neurosci.

[6] Abbott NJ, Ronnback L, Hansson E (2006). Astrocyte-endothelial interactions at the blood–brain barrier. Nat Rev Neurosci.

[7] Saade NE, Jabbur SJ (2008). Nociceptive behavior in animal models for peripheral neuropathy: spinal and supraspinal mechanisms. Prog Neurobiol.

[8] McMahon SB, Malcangio M (2009). Current challenges in glia-pain biology. Neuron.

[9] Vallejo R, Tilley DM, Vogel L, Benyamin R (2010). The role of glia and the immune system in the development and maintenance of neuropathic pain. Pain Pract.

[10] Dong H, Zhang X, Qian Y (2014). Mast cells and neuroinflammation. Med Sci Monit Basic Res.

[11] Jansson D, Rustenhoven J, Feng S, Hurley D, Oldfield RL, Bergin PS, Mee EW, Faull RL, Dragunow M (2014). A role for human brain pericytes in neuroinflammation. J Neuroinflammation.

[12] LeBleu VS, Taduri G, O'Connell J, Teng Y, Cooke VG, Woda C, Sugimoto H, Kalluri R (2013). Origin and function of myofibroblasts in kidney fibrosis. Nat Med.

[13] Scholz J, Woolf CJ (2007). The neuropathic pain triad: neurons, immune cells and glia. Nat Neurosci.

[14] Ji RR, Berta T, Nedergaard M (2013). Glia and pain: is chronic pain a gliopathy?. Pain.

[15] DeLeo JA, Tanga FY, Tawfik VL (2004). Neuroimmune activation and neuroinflammation in chronic pain and opioid tolerance/hyperalgesia. Neuroscientist.

[16] Milligan ED, Watkins LR (2009). Pathological and protective roles of glia in chronic pain. Nat Rev Neurosci.

[17] Tenorio G, Kulkarni A, Kerr BJ (2013). Resident glial cell activation in response to perispinal inflammation leads to acute changes in nociceptive sensitivity: implications for the generation of neuropathic pain. Pain.

[18] Koltzenburg M, Wall PD, McMahon SB (1999). Does the right side know what the left is doing?. Trends Neurosci.

[19] Blomstrand F, Khatibi S, Muyderman H, Hansson E, Olsson T, Ronnback L (1999). 5-Hydroxytryptamine and glutamate modulate velocity and extent of intercellular calcium signalling in hippocampal astroglial cells in primary cultures. Neuroscience.

[20] Berridge MJ, Bootman MD, Lipp P (1998). Calcium--a life and death signal. Nature.

[21] Zorec R, Araque A, Carmignoto G, Haydon PG, Verkhratsky A, Parpura V. Astroglial excitability and gliotransmission: an appraisal of Ca2+ as a signalling route. ASN Neuro 2012; 4. doi:10.1042/AN20110061. 10.1042/AN20110061 PMC3310306 22313347

[22] Head BP, Patel HH, Insel PA (1838). Interaction of membrane/lipid rafts with the cytoskeleton: impact on signaling and function: membrane/lipid rafts, mediators of cytoskeletal arrangement and cell signaling. Biochim Biophys Acta.

[23] Giaume C, McCarthy KD (1996). Control of gap-junctional communication in astrocytic networks. Trends Neurosci.

[24] Bennett MV, Garre JM, Orellana JA, Bukauskas FF, Nedergaard M, Saez JC (2012). Connexin and pannexin hemichannels in inflammatory responses of glia and neurons. Brain Res.

[25] Chen MJ, Kress B, Han X, Moll K, Peng W, Ji RR, Nedergaard M (2012). Astrocytic CX43 hemichannels and gap junctions play a crucial role in development of chronic neuropathic pain following spinal cord injury. Glia.

[26] Oda T, Iwasa M, Aihara T, Maeda Y, Narita A (2009). The nature of the globular- to fibrous-actin transition. Nature.

[27] Kimelberg HK, Macvicar BA, Sontheimer H (2006). Anion channels in astrocytes: biophysics, pharmacology, and function. Glia.

[28] Parpura V, Zorec R (2010). Gliotransmission: Exocytotic release from astrocytes. Brain Res Rev.

[29] Scemes E, Giaume C (2006). Astrocyte calcium waves: what they are and what they do. Glia.

[30] Cornell-Bell AH, Finkbeiner SM, Cooper MS, Smith SJ (1990). Glutamate induces calcium waves in cultured astrocytes: long-range glial signaling. Science.

[31] Lencesova L, O'Neill A, Resneck WG, Bloch RJ, Blaustein MP (2004). Plasma membrane-cytoskeleton-endoplasmic reticulum complexes in neurons and astrocytes. J Biol Chem.

[32] Liu X, Spicarova Z, Rydholm S, Li J, Brismar H, Aperia A (2008). Ankyrin B modulates the function of Na, K-ATPase/inositol 1,4,5-trisphosphate receptor signaling microdomain. J Biol Chem.

[33] Hansson E, Ronnback L (2003). Glial neuronal signaling in the central nervous system. FASEB J.

[34] Hansson E (2006). Could chronic pain and spread of pain sensation be induced and maintained by glial activation?. Acta Physiol (Oxf).

[35] Delbro D, Westerlund A, Bjorklund U, Hansson E (2009). In inflammatory reactive astrocytes co-cultured with brain endothelial cells nicotine-evoked Ca(2+) transients are attenuated due to interleukin-1beta release and rearrangement of actin filaments. Neuroscience.

[36] Cotrina ML, Lin JH, Alves-Rodrigues A, Liu S, Li J, Azmi-Ghadimi H, Kang J, Naus CC, Nedergaard M (1998). Connexins regulate calcium signaling by controlling ATP release. Proc Natl Acad Sci U S A.

[37] Edwards JR, Gibson WG (2010). A model for Ca2+ waves in networks of glial cells incorporating both intercellular and extracellular communication pathways. J Theor Biol.

[38] Sergeeva M, Ubl JJ, Reiser G (2000). Disruption of actin cytoskeleton in cultured rat astrocytes suppresses ATP- and bradykinin-induced [Ca(2+)](i) oscillations by reducing the coupling efficiency between Ca(2+) release, capacitative Ca(2+) entry, and store refilling. Neuroscience.

[39] Schreiner AE, Durry S, Aida T, Stock MC, Ruther U, Tanaka K, Rose CR, Kafitz KW (2014). Laminar and subcellular heterogeneity of GLAST and GLT-1 immunoreactivity in the developing postnatal mouse hippocampus. J Comp Neurol.

[40] Verkhratsky A, Nedergaard M, Hertz L (2014). Why are Astrocytes Important?. Neurochem Res.

[41] Muramatsu T, Uekusa T, Masaoka T, Saitoh M, Hashimoto S, Abiko Y, Jung HS, Shimono M (2008). Differential expression and localization of connexins 26 and 43 in the rat gingival epithelium. Arch Histol Cytol.

[42] Tu CL, Chang W, Bikle DD (2007). The role of the calcium sensing receptor in regulating intracellular calcium handling in human epidermal keratinocytes. J Invest Dermatol.

[43] Spohn D, Rossler OG, Philipp SE, Raubuch M, Kitajima S, Griesemer D, Hoth M, Thiel G (2010). Thapsigargin induces expression of activating transcription factor 3 in human keratinocytes involving Ca2+ ions and c-Jun N-terminal protein kinase. Mol Pharmacol.

[44] Takada H, Furuya K, Sokabe M (2014). Mechanosensitive ATP release from hemichannels and Ca(2)(+) influx through TRPC6 accelerate wound closure in keratinocytes. J Cell Sci.

[45] Barr TP, Albrecht PJ, Hou Q, Mongin AA, Strichartz GR, Rice FL (2013). Air-stimulated ATP release from keratinocytes occurs through connexin hemichannels. PLoS One.

[46] Genever PG, Maxfield SJ, Kennovin GD, Maltman J, Bowgen CJ, Raxworthy MJ, Skerry TM (1999). Evidence for a novel glutamate-mediated signaling pathway in keratinocytes. J Invest Dermatol.

[47] Tonon R, D'Andrea P (2000). Interleukin-1beta increases the functional expression of connexin 43 in articular chondrocytes: evidence for a Ca2 + −dependent mechanism. J Bone Miner Res.

[48] Mayan MD, Carpintero-Fernandez P, Gago-Fuentes R, Martinez-de-Ilarduya O, Wang HZ, Valiunas V, Brink P, Blanco FJ (2013). Human articular chondrocytes express multiple gap junction proteins: differential expression of connexins in normal and osteoarthritic cartilage. Am J Pathol.

[49] Vittur F, Grandolfo M, Fragonas E, Godeas C, Paoletti S, Pollesello P, Kvam BJ, Ruzzier F, Starc T, Mozrzymas JW (1994). Energy metabolism, replicative ability, intracellular calcium concentration, and ionic channels of horse articular chondrocytes. Exp Cell Res.

[50] Wilkins RJ, Fairfax TP, Davies ME, Muzyamba MC, Gibson JS (2003). Homeostasis of intracellular Ca2+ in equine chondrocytes: response to hypotonic shock. Equine Vet J.

[51] Zhang J, Zhang HY, Zhang M, Qiu ZY, Wu YP, Callaway DA, Jiang JX, Lu L, Jing L, Yang T, Wang MQ (2014). Connexin43 hemichannels mediate small molecule exchange between chondrocytes and matrix in biomechanically-stimulated temporomandibular joint cartilage. Osteoarthritis Cartilage.

[52] Chi SS, Rattner JB, Matyas JR (2004). Communication between paired chondrocytes in the superficial zone of articular cartilage. J Anat.

[53] Koolpe M, Benton HP (1997). Calcium-mobilizing purine receptors on the surface of mammalian articular chondrocytes. J Orthop Res.

[54] Piepoli T, Mennuni L, Zerbi S, Lanza M, Rovati LC, Caselli G (2009). Glutamate signaling in chondrocytes and the potential involvement of NMDA receptors in cell proliferation and inflammatory gene expression. Osteoarthritis Cartilage.

[55] Stains JP, Civitelli R (2005). Gap junctions in skeletal development and function. Biochimica Et Biophysica Acta-Biomembranes.

[56] Jorgensen NR, Henriksen Z, Brot C, Eriksen EF, Sorensen OH, Civitelli R, Steinberg TH (2000). Human osteoblastic cells propagate intercellular calcium signals by two different mechanisms. J Bone Miner Res.

[57] Gupta A, Niger C, Buo AM, Eidelman ER, Chen RJ, Stains JP (2014). Connexin43 enhances the expression of osteoarthritis-associated genes in synovial fibroblasts in culture. BMC Musculoskelet Disord.

[58] Wall ME, Banes AJ (2005). Early responses to mechanical load in tendon: role for calcium signaling, gap junctions and intercellular communication. J Musculoskelet Neuronal Interact.

[59] Maeda E, Ye S, Wang W, Bader DL, Knight MM, Lee DA (2012). Gap junction permeability between tenocytes within tendon fascicles is suppressed by tensile loading. Biomech Model Mechanobiol.

[60] Waggett AD, Benjamin M, Ralphs JR (2006). Connexin 32 and 43 gap junctions differentially modulate tenocyte response to cyclic mechanical load. Eur J Cell Biol.

[61] Wall ME, Otey C, Qi J, Banes AJ (2007). Connexin 43 is localized with actin in tenocytes. Cell Motil Cytoskeleton.

[62] Rohr S (2011). Cardiac fibroblasts in cell culture systems: myofibroblasts all along?. J Cardiovasc Pharmacol.

[63] Askar SF, Bingen BO, Swildens J, Ypey DL, van der Laarse A, Atsma DE, Zeppenfeld K, Schalij MJ, de Vries AA, Pijnappels DA (2012). Connexin43 silencing in myofibroblasts prevents arrhythmias in myocardial cultures: role of maximal diastolic potential. Cardiovasc Res.

[64] Liang W, McDonald P, McManus B, van Breemen C, Wang X (2008). Characteristics of agonist-induced Ca2+ responses in diseased human valvular myofibroblasts. Proc West Pharmacol Soc.

[65] Riches K, Hettiarachchi NT, Porter KE, Peers C (2010). Hypoxic remodelling of Ca2+ stores does not alter human cardiac myofibroblast invasion. Biochem Biophys Res Commun.

[66] Zeng QC, Guo Y, Liu L, Zhang XZ, Li RX, Zhang CQ, Hao QX, Shi CH, Wu JM, Guan J (2013). Cardiac fibroblast-derived extracellular matrix produced in vitro stimulates growth and metabolism of cultured ventricular cells. Int Heart J.

[67] Gaspers LD, Thomas AP (2005). Calcium signaling in liver. Cell Calcium.

[68] Balasubramaniyan V, Dhar DK, Warner AE, Vivien Li WY, Amiri AF, Bright B, Mookerjee RP, Davies NA, Becker DL, Jalan R (2013). Importance of Connexin-43 based gap junction in cirrhosis and acute-on-chronic liver failure. J Hepatol.

[69] Schlosser SF, Burgstahler AD, Nathanson MH (1996). Isolated rat hepatocytes can signal to other hepatocytes and bile duct cells by release of nucleotides. Proc Natl Acad Sci U S A.

[70] Zimmermann B, Walz B (1999). The mechanism mediating regenerative intercellular Ca2+ waves in the blowfly salivary gland. Embo Journal.

[71] Petersen OH, Tepikin AV (2008). Polarized calcium signaling in exocrine gland cells. Annu Rev Physiol.

[72] Kielian T (2006). Toll-like receptors in central nervous system glial inflammation and homeostasis. J Neurosci Res.

[73] Forshammar J, Block L, Lundborg C, Biber B, Hansson E (2011). Naloxone and ouabain in ultralow concentrations restore Na+/K + −ATPase and cytoskeleton in lipopolysaccharide-treated astrocytes. J Biol Chem.

[74] Hutchinson MR, Zhang Y, Brown K, Coats BD, Shridhar M, Sholar PW, Patel SJ, Crysdale NY, Harrison JA, Maier SF (2008). Non-stereoselective reversal of neuropathic pain by naloxone and naltrexone: involvement of toll-like receptor 4 (TLR4). Eur J Neurosci.

[75] Block L, Forshammar J, Westerlund A, Bjorklund U, Lundborg C, Biber B, Hansson E (2012). Naloxone in ultralow concentration restores endomorphin-1-evoked Ca(2)(+) signaling in lipopolysaccharide pretreated astrocytes. Neuroscience.

[76] Gorina R, Font-Nieves M, Marquez-Kisinousky L, Santalucia T, Planas AM (2011). Astrocyte TLR4 activation induces a proinflammatory environment through the interplay between MyD88-dependent NFkappaB signaling, MAPK, and Jak1/Stat1 pathways. Glia.

[77] Kleveta G, Borzecka K, Zdioruk M, Czerkies M, Kuberczyk H, Sybirna N, Sobota A, Kwiatkowska K (2012). LPS induces phosphorylation of actin-regulatory proteins leading to actin reassembly and macrophage motility. J Cell Biochem.

[78] Arraes SM, Freitas MS, da Silva SV, de Paula Neto HA, Alves-Filho JC, Auxiliadora Martins M, Basile-Filho A, Tavares-Murta BM, Barja-Fidalgo C, Cunha FQ (2006). Impaired neutrophil chemotaxis in sepsis associates with GRK expression and inhibition of actin assembly and tyrosine phosphorylation. Blood.

[79] Karpuk N, Burkovetskaya M, Fritz T, Angle A, Kielian T (2011). Neuroinflammation leads to region-dependent alterations in astrocyte gap junction communication and hemichannel activity. J Neurosci.

[80] Gegelashvili G, Schousboe A (1997). High affinity glutamate transporters: regulation of expression and activity. Mol Pharmacol.

[81] Takaki J, Fujimori K, Miura M, Suzuki T, Sekino Y, Sato K (2012). L-glutamate released from activated microglia downregulates astrocytic L-glutamate transporter expression in neuroinflammation: the ‘collusion’ hypothesis for increased extracellular L-glutamate concentration in neuroinflammation. J Neuroinflammation.

[82] Hansson E, Westerlund A, Bjorklund U, Olsson T (2008). mu-Opioid agonists inhibit the enhanced intracellular Ca(2+) responses in inflammatory activated astrocytes co-cultured with brain endothelial cells. Neuroscience.

[83] Lundborg C, Hahn-Zoric M, Biber B, Hansson E (2010). Glial cell line-derived neurotrophic factor is increased in cerebrospinal fluid but decreased in blood during long-term pain. J Neuroimmunol.

[84] Heppner FL, Ransohoff RM, Becher B (2015). Immune attack: the role of inflammation in Alzheimer disease. Nat Rev Neurosci.

[85] Herrero MT, Estrada C, Maatouk L, Vyas S (2015). Inflammation in Parkinson’s disease: role of glucocorticoids. Front Neuroanat.

[86] Pihl-Jensen G, Tsakiri A, Frederiksen JL (2015). Statin treatment in multiple sclerosis: a systematic review and meta-analysis. CNS Drugs.

[87] Sofroniew MV (2015). Astrocyte barriers to neurotoxic inflammation. Nat Rev Neurosci.

[88] Chen L, Guo S, Ranzer MJ, DiPietro LA (2013). Toll-like receptor 4 has an essential role in early skin wound healing. J Invest Dermatol.

[89] Portou MJ, Baker D, Abraham D, Tsui J. The innate immune system, toll-like receptors and dermal wound healing: A review. Vascul Pharmacol. 2015. doi:10.1016/j.vph.2015.02.007. 10.1016/j.vph.2015.02.007 25869514

[90] Ushio H, Nohara K, Fujimaki H (1999). Effect of environmental pollutants on the production of pro-inflammatory cytokines by normal human dermal keratinocytes. Toxicol Lett.

[91] Lorenz W, Buhrmann C, Mobasheri A, Lueders C, Shakibaei M (2013). Bacterial lipopolysaccharides form procollagen-endotoxin complexes that trigger cartilage inflammation and degeneration: implications for the development of rheumatoid arthritis. Arthritis Res Ther.

[92] Liu L, Gu H, Liu H, Jiao Y, Li K, Zhao Y, An L, Yang J (2014). Protective effect of resveratrol against IL-1beta-induced inflammatory response on human osteoarthritic chondrocytes partly via the TLR4/MyD88/NF-kappaB signaling pathway: an “in vitro study”. Int J Mol Sci.

[93] McNearney TA, Ma Y, Chen Y, Taglialatela G, Yin H, Zhang WR, Westlund KN (2010). A peripheral neuroimmune link: glutamate agonists upregulate NMDA NR1 receptor mRNA and protein, vimentin, TNF-alpha, and RANTES in cultured human synoviocytes. Am J Physiol Regul Integr Comp Physiol.

[94] Jean YH, Wen ZH, Chang YC, Hsieh SP, Lin JD, Tang CC, Chen WF, Chou AK, Wong CS (2008). Increase in excitatory amino acid concentration and transporters expression in osteoarthritic knees of anterior cruciate ligament transected rabbits. Osteoarthritis and Cartilage.

[95] Bonnet CS, Williams AS, Gilbert SJ, Harvey AK, Evans BA, Mason DJ (2015). AMPA/kainate glutamate receptors contribute to inflammation, degeneration and pain related behaviour in inflammatory stages of arthritis. Ann Rheum Dis.

[96] Vincenzi F, Targa M, Corciulo C, Gessi S, Merighi S, Setti S, Cadossi R, Goldring MB, Borea PA, Varani K (2013). Pulsed electromagnetic fields increased the anti-inflammatory effect of A(2)A and A(3) adenosine receptors in human T/C-28a2 chondrocytes and hFOB 1.19 osteoblasts. PLoS One.

[97] Lindner D, Zietsch C, Tank J, Sossalla S, Fluschnik N, Hinrichs S, Maier L, Poller W, Blankenberg S, Schultheiss HP (2014). Cardiac fibroblasts support cardiac inflammation in heart failure. Basic Res Cardiol.

[98] Javkhlan P, Hiroshima Y, Azlina A, Hasegawa T, Yao CJ, Akamatsu T, Kido J, Nagata T, Hosoi K (2011). Lipopolysaccharide-Mediated Induction of Calprotectin in the Submandibular and Parotid Glands of Mice. Inflammation.

[99] Ingman WV, Glynn DJ, Hutchinson MR (2014). Inflammatory mediators in mastitis and lactation insufficiency. J Mammary Gland Biol Neoplasia.

[100] Redfern RL, Patel N, Hanlon S, Farley W, Gondo M, Pflugfelder SC, McDermott AM (2013). Toll-Like Receptor Expression and Activation in Mice with Experimental Dry Eye. Investigative Ophthalmology & Visual Science.

[101] Zare-Bidaki M, Tsukiyama-Kohara K, Arababadi MK (2014). Toll-like receptor 4 and hepatitis B infection: molecular mechanisms and pathogenesis. Viral Immunol.

[102] Perros F, Lambrecht BN, Hammad H (2011). TLR4 signalling in pulmonary stromal cells is critical for inflammation and immunity in the airways. Respir Res.

[103] Greenberg AS, Obin MS (2006). Obesity and the role of adipose tissue in inflammation and metabolism. Am J Clin Nutr.

[104] Hotamisligil GS (2006). Inflammation and metabolic disorders. Nature.

[105] Bleau C, Karelis AD, St-Pierre DH, Lamontagne L. Crosstalk between intestinal microbiota, adipose tissue and skeletal muscle as an early event in systemic low-grade inflammation and the development of obesity and diabetes. Diabetes Metab Res Rev. 2014. doi:10.1002/dmrr.2617. 10.1002/dmrr.2617 25352002

[106] Verdile G, Fuller SJ, Martins RN (2015). The role of type 2 diabetes in neurodegeneration. Neurobiol Dis.

[107] Prasad M, Hermann J, Gabriel SE, Weyand CM, Mulvagh S, Mankad R, Oh JK, Matteson EL, Lerman A (2014). Cardiorheumatology: cardiac involvement in systemic rheumatic disease. Nat Rev Cardiol.

[108] O'Conor CJ, Leddy HA, Benefield HC, Liedtke WB, Guilak F (2014). TRPV4-mediated mechanotransduction regulates the metabolic response of chondrocytes to dynamic loading. Proc Natl Acad Sci U S A.

[109] Verkhratsky A, Reyes RC, Parpura V (2014). TRP channels coordinate ion signaling in astroglia. Rev Physiol Biochem Pharmacol.

[110] Smani T, Shapovalov G, Skryma R, Prevarskaya N, Rosado JA (1853). Functional and physiopathological implications of TRP channels. Biochim Biophys Acta.

[111] Zhao P, Lieu T, Barlow N, Sostegni S, Haerteis S, Korbmacher C, Liedtke W, Jimenez-Vargas NN, Vanner SJ, Bunnett NW (2015). Neutrophil Elastase Activates Protease-activated Receptor-2 (PAR2) and Transient Receptor Potential Vanilloid 4 (TRPV4) to Cause Inflammation and Pain. J Biol Chem.

[112] Fernandes ES, Awal S, Karadaghi R, Brain SD (2013). TRP receptors in arthritis, gaining knowledge for translation from experimental models. The open pain journal.

[113] Yang CA, Chiang BL (2015). Inflammasomes and human autoimmunity: A comprehensive review. J Autoimmun.

[114] Hansson E (2014). Actin Filament Reorganization in Astrocyte Networks is a Key Functional Step in Neuroinflammation Resulting in Persistent Pain: Novel Findings on Network Restoration. Neurochem Res.

[115] Jancalek R (2011). Signaling mechanisms in mirror image pain pathogenesis. Ann Neurosci.

